# Optimizing laser-driven proton acceleration from overdense targets

**DOI:** 10.1038/srep29402

**Published:** 2016-07-20

**Authors:** A. Stockem Novo, M. C. Kaluza, R. A. Fonseca, L. O. Silva

**Affiliations:** 1 Institut für Theoretische Physik, Lehrstuhl IV: Weltraum- & Astrophysik, Ruhr-Universität, Bochum, Germany; 2 Institute of Optics and Quantum Electronics, University of Jena, Germany; 3 Helmholtz-Institute Jena, Germany; 4 GoLP/Instituto de Plasmas e Fusão Nuclear - Laboratório Associado, Instituto Superior Técnico, Universidade de Lisboa, Lisboa, Portugal; 5 ISCTE Instituto Universitário Lisboa, Portugal

## Abstract

We demonstrate how to tune the main ion acceleration mechanism in laser-plasma interactions to collisionless shock acceleration, thus achieving control over the final ion beam properties (e. g. maximum energy, divergence, number of accelerated ions). We investigate this technique with three-dimensional particle-in-cell simulations and illustrate a possible experimental realisation. The setup consists of an isolated solid density target, which is preheated by a first laser pulse to initiate target expansion, and a second one to trigger acceleration. The timing between the two laser pulses allows to access all ion acceleration regimes, ranging from target normal sheath acceleration, to hole boring and collisionless shock acceleration. We further demonstrate that the most energetic ions are produced by collisionless shock acceleration, if the target density is near-critical, *n*_*e*_ ≈ 0.5 *n*_*cr*_. A scaling of the laser power shows that 100 MeV protons may be achieved in the PW range.

Laser-acceleration of ions is currently a topic of high relevance for a wide range of possible applications^1^,^2^,^3^ and associated with many fundamental processes in the laboratory and in astrophysics^4^,^5^. In particular, ion acceleration processes in laser-driven scenarios are being studied to realise compact laser-based particle accelerators capable of producing ion beams of high quality. Understanding and controlling the different ion acceleration mechanisms^6^,^7^,^8^,^9^,^10^,^11^, as well as their interplay, is of paramount importance for future developments. Therefore, it is critical to identify scenarios where this control and detailed analysis can be performed, both from a fundamental perspective but also for experimental conditions now being pursued^12^,^13^,^14^,^15^,^16^,^17^.

Inspired by previous work on the formation of shock shells in nano plasmas^18^,^19^, we propose a laser irradiation of a macroscopic finite size target to tailor the phase space of the accelerated ions by modifying the target density in an appropriate way. In this paper, we show that the shaping of the ion phase space can be achieved by triggering and combining different acceleration mechanisms. By using a first laser pulse for a pre-heating stage and a second pulse to actually drive the acceleration process^19^,^20^, one initial target setup can be used to enter the different acceleration regimes, by changing the delay between the two pulses and the intensity of the first pulse.

A first low-intensity laser pulse with normalised laser vector potential *a*_0_ = *eA*_0_/*m*_*e*_*c*^2^ < 1, where *e* is the electron charge, *A*_0_ the laser vector potential, *m*_*e*_ the electron mass and *c* the speed of light, irradiates a spherical solid density target (Fig. 1a). The electron energy increases which is followed by a hydrodynamic expansion of the target (Fig. 1b) with the ion sound speed 
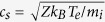
, where Z is the ion charge, k_B_ the Boltzmann constant, T_e_ the electron temperature and m_i_ the ion mass^21^,^22^. For laser intensities below 10^16^ W/cm^2^, corresponding to 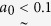, collisional energy absorption processes dominate (inverse bremsstrahlung), while for larger intensities collisionless processes (resonance absorption, Brunel heating) are more important. For simplicity, a uniform target density *n*_0_ after expansion is assumed with a radius increase from R* to *R* by a factor 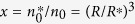. The expansion time at which the target density has been reduced by a factor *x* is then given by


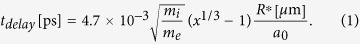


We expressed the temperature in terms of the laser potential according to ponderomotive heating, 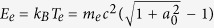
23 for a small argument expansion 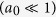. This model is rather simple, e. g. neglecting the density ramps at the target surface or non-uniform target heating and expansion, but it does capture the main questions addressed in this paper.

The actual ion acceleration is then triggered by a second high intensity laser pulse 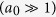 incident on the pre-heated target (Fig. 1c,d). The dominant acceleration process depends on the target density at the time of arrival of the main pulse. Thus, the correct timing of the interaction of the second laser pulse with the target is critical to guarantee that collisionless shock acceleration can prevail over target-normal-sheath-acceleration (TNSA), which is always present at the surface of overdense targets (compare also the 1D and 2D simulations in refs 24 and 25 which show the efficiency of collisionless shock acceleration towards TNSA). We now review the main acceleration mechanisms that can play a role in this configuration.

Typically in the TNSA scheme, *μ*m scale overdense targets are irradiated by a high-intensity laser pulse. The laser cannot penetrate the target beyond densities greater than the relativistic critical density 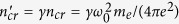, with the correction due to the relativistic gamma factor of the electrons 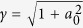, and the laser frequency *ω*_0_. The laser pulse is partially absorbed at the target front surface accelerating electrons to relativistic energies due to ponderomotive heating. These hot electrons propagate across the target and escape at the rear side. The subsequent electrostatic field at the target rear side accelerates the positive ions along the target normal direction to an energy of roughly *E*_*i*,*TNSA*_ = *ZE*_*e*_^9^,^23^. The highest proton energies experimentally achieved with TNSA are 85 MeV cite (F. Wagner *et al*., PRL 116 205002, 2016)^8^. TNSA is very sensitive to the particular laser and target configurations and the initial setup^26^. The ion energy spectrum can typically be described by a thermal-like exponentially decreasing distribution which leads to only a small number of accelerated particles close to the maximum energy cutoff.

Even if the laser cannot propagate through overdense targets, the ponderomotive force is strong enough to push the electrons at the target front surface. As a consequence, and since the ions remain quasi-stationary due to their higher inertia, a quasi-static electric field is created at the front surface oriented into the target and propagating at the hole boring velocity 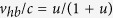 with 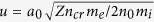
9,23. Ions reflected from the hole boring potential will acquire a velocity into the target of approximately 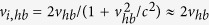, which determines the scaling for hole boring acceleration (HBA).

In near-critical density targets, if the hole boring velocity is larger than the ion sound speed, *v*_*hb*_ > *c*_*s*_, which is equivalent to the condition *n*_0_/*n*_*cr*_ < *a*_0_ for 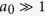, the propagation of a shock can be observed ahead of the hole boring compression with a velocity 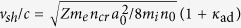 and the adiabatic coefficient *κ*_ad_ = 5/3 from ideal 3D gas theory^27^. A lower limit is obtained from the condition that the density of the downstream (the compressed plasma moving into the target) has to be larger than the density of the upstream (the plasma target at rest with initial density *n*_0_), 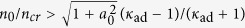
27. Also in this case, the ions are picked up by the propagating potential and accelerated with approximately twice the shock velocity to 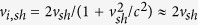, resulting in an energy *E*_*i*,*sh*_ = *m*_*i*_*c*^2^(*γ*_*i*,*sh*_ − 1) with 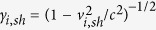 12. If the shock velocity is non-relativistic and 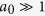, the final ion energy can be approximated as


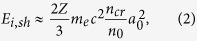


cf.^28^,^29^. Introducing *y* = *n*_0_/*γn*_*cr*_ ≈ *n*_0_/(*a*_0_*n*_*cr*_), the ion energy is given as


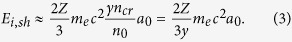


We note that in general *v*_*sh*_ > *v*_*hb*_ and thus electrostatic collisionless shock acceleration (CSA) will always lead to higher ion energies than HBA.

If the target density is further decreased to underdense conditions, i.e. 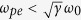, the laser can penetrate the target. However, if the target is thin enough and the laser pulse is long enough that the laser can break through the rear surface, a significant increase in energy is expected from an enhanced TNSA effect, which we also observe in our simulations.

## Results

**Figure 2:**
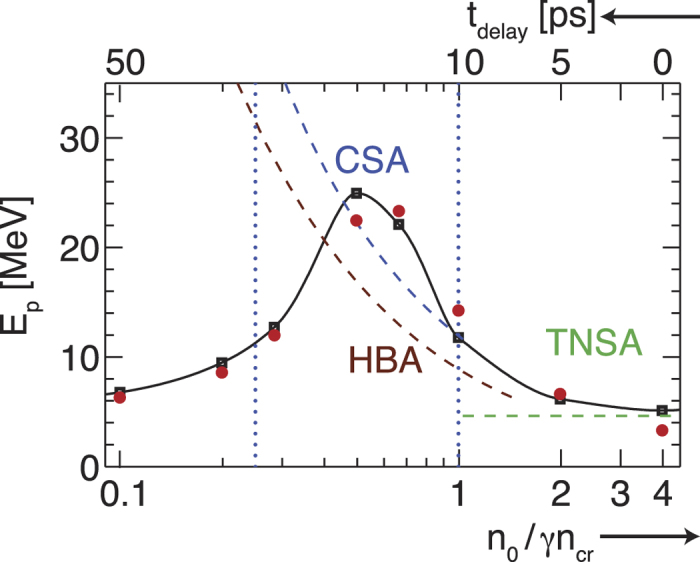
Maximum proton energies expected from theory as function of the relativistic target density for *a*_0_ = 10 due to different acceleration mechanisms: target normal sheath acceleration (green), collisionless shock acceleration (blue), hole boring (brown). The time axis corresponds to the time delay between the heating pulse with *a*_0_ = 0.1 initiating a hydrodynamic expansion and the second laser pulse. The maximum proton energies in the simulations measured inside the target are compared for a cubed (black squares) and a spherical target (red dots).

This framework can now be used to interpret and to explore the proposed scenario of ion acceleration. We illustrate this set-up with full scale three dimensional particle-in-cell (PIC) simulations modelling the interaction of a laser pulse with a macroscopic finite size target. The simulations were performed with the PIC code OSIRIS^30^,^31^. We model a full realistic experimental setup in three dimensions, which allows us to determine quantitatively all the relevant beam parameters, i.e. (maximum) proton energy, the spectrum, and distributions. The initial setup consists of a spherical target with R* = 10 *μ*m being the radius of the sphere, with homogeneous initial density 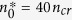 corresponding to a frozen hydrogen pellet with *m*_*p*_/*m*_*e*_ = 1836. Different target shapes (spherical and cubic) and initial radii have also been tested maintaining the same volume and total number of particles. Additionally, a cylindrical jet with infinite extension along an axis perpendicular to the laser propagation direction has been simulated. For simplicity, the simulation starts with the interaction of the second high intensity laser pulse, which is injected along *x*_1_ with normalised intensity in the range *a*_0_ = 2–47 with a transverse Gaussian profile, and with linear polarisation in *x*_2_ direction. We also modelled the full set-up, including the interaction of the first laser pulse with the cold, spherical target, leading to a density gradient in the target density, to the interaction of a second laser pulse with the self-consistently expanded target. We found that the observed lower density at the target surface leads only to a small shift on the reference density for the different regimes (cf. Fig. 2). Since the impact of the inclusion of the self-consistent expansion on the dominant acceleration mechanism is very low, we adopted the simplest model possible, i.e. a spherical target with uniform density characterised by just the uniform density parameter.

In our simulations, the parameters of the second laser pulse are the laser wavelength *λ*_0_ = 1 *μ*m = 2*π* *c*/*ω*_0_ with a pulse length 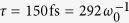, focused on the target front side. The spot size is *w*_0_ = 3.5 *μ*m for *a*_0_ = 10 and is scaled as *a*_0_*w*_0_ = const. according to 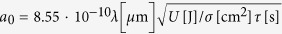 with an approximated focal area of 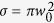. These are typical parameters of current laser systems^32^. The cubic simulation box was adapted to the increasing target volume with a maximum box length of *L* = 855 *c*/*ω*_0_ and 2760 cells per dimension with two particles per cell. The cell size was chosen to guarantee a sampling frequency of at least two points per characteristic wavelength in the overdense plasma, 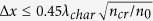. For a detailed list of the parameters, see Tables 1, 2, 3.

The central results of the parameter scan with constant *a*_0_ = 10 (Table 1) performed to illustrate the ion acceleration set-up are presented in Fig. 2. The highest energies in the simulations match with the theoretical prediction. TNSA dominates in the regime of highly overdense targets, which in this setup generates protons with ≈5 MeV maximum energy. The results seem to be independent of the actual target shape (cube or sphere). The maximum proton energy is observed at near-critical density due to CSA and is highest (*E*_*p*_ ≈ 25 MeV) for a target density *n*_0_/*γn*_*cr*_ ≈ 0.5. We observe the same trend as in Fig. 2, with a peak energy for shock acceleration at *n*_0_/*γn*_*cr*_ ≈ 0.5, if the initial density is kept fixed and the laser intensity is varied (parameters given in Table 3). In the supplementary material the proton phase spaces of representative cases are shown in Fig. S1.

## Discussion

A detailed analysis of the overdense regime *n*_0_/*γn*_*cr*_ ≥ 0.5 shows a strong density compression propagating through the target. In the pure TNSA-regime *n*_0_/*γn*_*cr*_ > 2, the associated electric field is not strong enough to accelerate the ions inside the target and acceleration occurs mainly at the target rear surface (see Fig. S1a).

**Figure 3:**
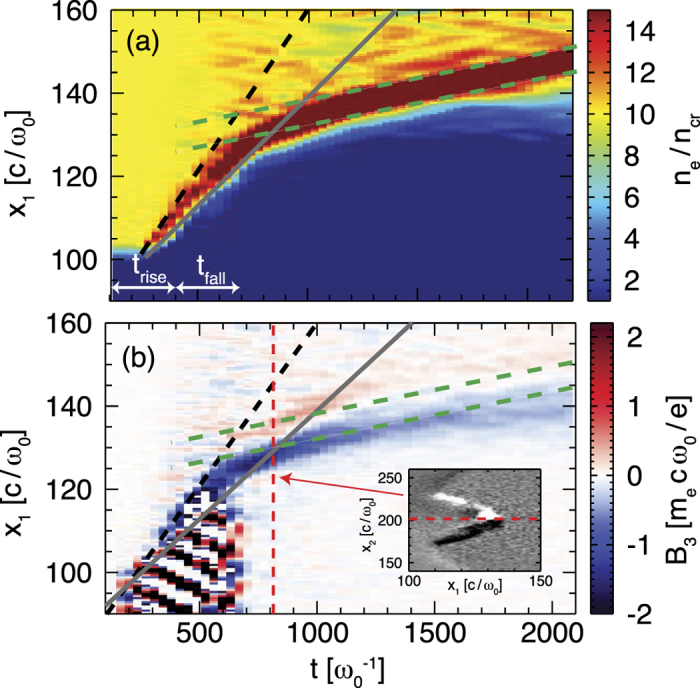
Density (**a**) and magnetic field (**b**) evolution along *x*_1_ at *x*_2_ = *x*_3_ = 200 *c*/*ω*_0_ for initial target density *n*_0_/*γn*_*cr*_ = 1, laser intensity *a*_0_ = 10 and 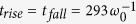. The grey and black lines show the hole boring velocity, *v*_*hb*_, and shock velocity, *v*_*sh*_, respectively. The green dashed lines have a slope *v* = 0.01 c and the inset shows *B*_3_ at *tω*_0_ = 806.

In the case of near-critical densities 0.5 ≤ *n*_0_/*γn*_*cr*_ ≤ 2 a strong peak of the electric field is formed at the target front which is co-moving with the region of density compression. It picks up the upstream protons and accelerates them to approximately twice the peak velocity, Eq. (3). This is illustrated in Fig. 3(a) for *n*_0_/(*γn*_*cr*_) = 1, where two velocities can be clearly distinguished, the hole boring velocity *v*_*hb*_ = 0.0522 *c* and the shock velocity *v*_*sh*_ = 0.0696 *c* > *v*_*hb*_. Due to the thermal expansion of the target, the protons have an additional velocity *v*_0_ = 0.02 *c*, which slightly increases their final velocity to ≈2 *v*_*sh*_ + *v*_0_. The electrons are heated to approximately 0.8 MeV, thus the shock Mach number is *M* = *v*_*sh*_/*c*_*s*_ ≈ 2.4. The bunch of accelerated particles can be clearly identified in the ion phase space in Fig. S1(b), where collisionless shock acceleration at the target front dominates over TNSA at the target rear surface.

A collimating azimuthal magnetic field is built-up, see inset of Fig. 3(b) showing *B*_3_ (a similar picture is obtained for *B*_2_ with *B*_2_ and *B*_3_ being the components of the azimuthal magnetic field). It is related to the balance of the hot electron beam through the target by a fast cold return current *I* = *r*_*e*_*cB*/2 = 12 *m*_*e*_*c*^3^/*e* with a maximum Larmor radius of *r*_*e*_ = 16 *c*/*ω*_0_ for the hottest electrons and a maximum magnetic field strength *B* ≈ 1.5 *m*_*e*_*cω*_0_/*e*^33^,^34^. The magnetic field guides the electrons in the direction of the back of the target at a constant velocity *v* = 0.01 *c* after the laser is off, see Fig. 3(b). We stress that this mechanism to enhance and to guarantee the conditions for shock propagation was not previously identified in the literature. The density compression is maintained by the collimation of the electron beam.

We note that for densities 0.5 < *n*_0_/*γn*_*cr*_ < 1 the compression at the target surface due to the radiation pressure of the laser makes the target opaque to the laser, and shock acceleration inside the target is still observed. In the underdense scenario, *n*_0_/*γn*_*cr*_ < 0.5, this compression is not strong enough and the laser can propagate. Proton acceleration inside the target is rather weak in this case.

**Figure 4:**
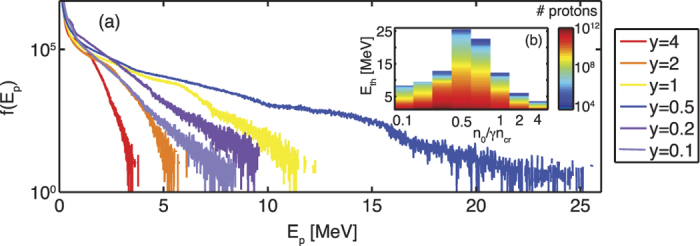
(**a**) Proton energy spectra for *a*_0_ = 10 at *t* = 0.8 ps and varying target densities given in *y* = *n*_0_/*γn*_*cr*_. (**b**) Number of protons with energy above a threshold energy *E*_*th*_ vs. threshold energy and target density *n*_0_/*γn*_*cr*_.

**Figure 5:**
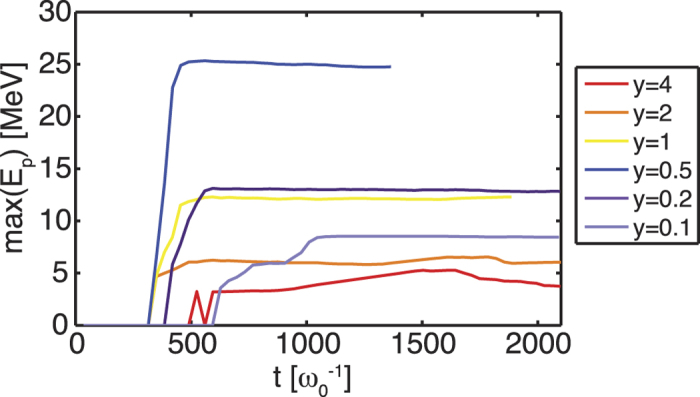
Maximum proton energy vs. time.

In addition to the ion energy, it is also important to consider the total number of accelerated protons to the relevant energy ranges. Fully quantitative results can only be obtained in three dimensional simulations. Figure 4 shows, for *a*_0_ = 10 and varying target density, the proton spectra and the number of protons with energies above a threshold energy *E*_*th*_. The clear dominance of shock acceleration can also be recognised here. There are still more than 10^8^ protons at energies *E* > 15 MeV (see inset in Fig. 4), which would guarantee a sufficient number of particles for many applications^35^,^36^,^37^. See also the comparison of the beam divergence (Fig. S2) which shows the advantage of collisionless shock acceleration towards TNSA. The TNSA case shows an almost uniform distribution of the protons while the CSA case shows a narrow distribution at low angles. Figure 5 shows that the proton energy is saturated during the quasi-steady state of shock propagation. We calculated the energy conversion efficiency, *E*_*kin*,*total*_/*E*_*laser*_, from the laser to protons with energies >4 MeV being accelerated within an angle *θ* < 10° where *θ* is defined as tan *θ* = *p*_⊥_/*p*_‖_ with *p*_⊥_ and *p*_‖_ the transverse and parallel components of the proton momenta, respectively. The energy conversion is 6–7 orders of magnitude higher for CSA than for pure TNSA.

It has to be mentioned that in 3D simulations the maximum energies are significantly smaller and overall TNSA acceleration is much weaker than in a comparable 2D run as was previously clarified in^38^. This is due to the different radial dependence of the accelerating electric field and the lower efficiency of the heating mechanism in the case of a linearly polarised laser.

**Figure 6:**
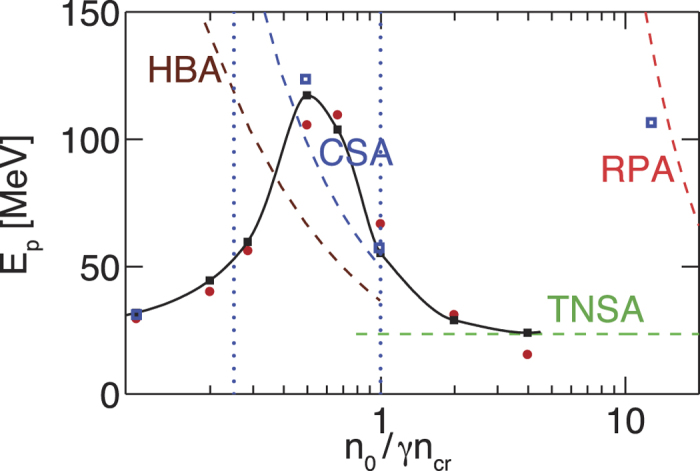
Maximum proton energy vs. varying target density *n*_0_*γn*_*cr*_: Simulation with 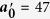 (blue squares) compared to case *a*_0_ = 10 (cf. Fig. 2) showing 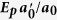.

We also tested the robustness of our study to varying the laser intensity and pulse length, also with a detailed analysis of the PW regime for 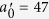 and 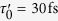, parameters see Table 2. We found that the discussion presented before holds, showing a similar scaling behaviour with the same dominant acceleration mechanisms as in the case for *a*_0_ = 10, see Fig. 6. The maximum proton energy is increased by a factor 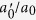 according to Eq. 3. (Note: We also explored the regime of radiation pressure acceleration (RPA) for highly compressed and thin targets. The target is destroyed and almost all the ions are accelerated, see Fig. S1c). The overall higher proton energy has its maximum *E*_*p*_ = 124 MeV at *n*_0_/*γn*_*cr*_ ≈ 0.5 in the overdense regime due to CSA. Thus, this scheme seems to be robust in order to explore the different acceleration mechanisms and energy scalings at higher laser intensities and is promising for achieving proton energies above 100 MeV in a realistic experimental setup with 10 PW laser systems.

In conclusion, we proposed a scheme that allows to test and to tune the dominant process for ion acceleration. An initially highly opaque spherical target is heated by a low intensity laser pulse leading to a reduction of the target density by a hydrodynamic expansion. By timing a second, intense driver pulse, the acceleration process is initiated. Our fully relativistic 3D particle-in-cell simulations of a realistic experimental setup show a maximum proton energy close to critical density, *n*_0_/*γn*_*cr*_ = 0.5, due to collisionless shock acceleration.

Collisionless shock acceleration also shows a clear dominance when the total number of accelerated protons and the beam divergence are considered. The highly collimated acceleration of particles along the shock propagation direction, due to the self-generated collimating magnetic field, guarantees a high quality of the beam. Our results thus indicate the fully quantitative path to laser-driven acceleration in order to achieve high energy and high quality beams.

## Additional Information

**How to cite this article**: Novo, A. S. *et al*. Optimizing laser-driven proton acceleration from overdense targets. *Sci. Rep.* **6**, 29402; doi: 10.1038/srep29402 (2016).

## Supplementary Material

Supplementary Information

## Figures and Tables

**Figure 1:**
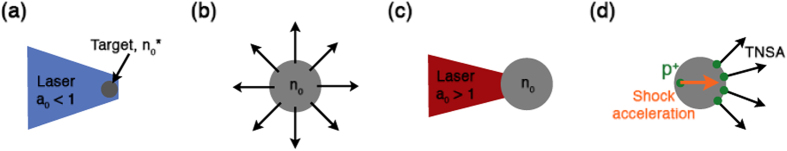
Simulation setup: (**a**) The high-density target is irradiated by a laser with large focal area (**b**) leading to hydrodynamic expansion of the target. (**c**) A second, stronger laser pulse launches (**d**) the acceleration process.

**Table 1:** Simulations with *a*_0_ = 10: Density after hydrodynamic expansion *n*_0_/*γn*_*cr*_, radius of the sphere *R* or alternative box length of the cube *L*_*c*_, simulation box length *L*_*box*_ and simulation cell size Δ*x* for a laser wavelength *λ* = 1 *μ*m.

***n***_**0**_**/*****γn***_***cr***_	***R ***[***μ*****m]**	***L***_***c***_*** ***[***μ*****m]**	***L***_***box***_*** ***[***μ*****m]**	**Δ*****x ***[***μ*****m]**
4	10	16	39.81	0.011
2	25	20	50.16	0.016
1	32	25	63.19	0.023
0.67	36	29	72.34	0.028
0.5	40	32	79.62	0.032
0.28	48	39	95.95	0.042
0.2	54	44	108.06	0.049
0.1	68	55	136.14	0.049

**Table 2:** Simulations with *a*_0_ = 47: Density after hydrodynamic expansion *n*_0_/*γn*_*cr*_, radius of the sphere *R* or alternative box length of the cube *L*_*c*_, simulation box length *L*_*box*_ and simulation cell size Δ*x* for a laser wavelength *λ* = 1*μ*m.

***n***_**0**_**/*****γn***_***cr***_	***L***_***c***_** [*****μ*****m]**	***L***_***box***_*** ***[***μ*****m]**	**Δ*****x***** [*****μ*****m]**
0.1	10.21	31.85	0.0433
0.5	2.08	23.89	0.0199
1	1.02	15.92	0.0143
13	0.08	7.99	0.0040

**Table 3:** Simulations with *n*_0_/*n*_*cr*_ = 6.7, box length *L*_*box*_ = 72.29 *μ*m, cell size Δ*x* = 0.0277 *μ*m and sphere radius *R* = 18.15 *μ*m: Normalised laser potential *a*_0_ and spot size *w*_0_ in SI- and normalised units.

***a***_**0**_	***w***_**0**_** [*****μ*****m]**	***w***_**0**_** [*****c*****/*****w***_**0**_]
2	17.50	109.90
4	8.75	54.95
6	5.83	36.63
8	4.38	27.48
10	3.50	21.98
12	2.92	18.32
18	1.94	12.21
28	1.25	7.85
