# Supplementary material for: Optimizing laser-driven proton acceleration from overdense targets
Source: Sci Rep. 2016 Jul 20;6:29402. doi: 10.1038/srep29402 (PMC4951642; doi:10.1038/srep29402)
Supplement: Supplementary Information [file srep29402-s1.pdf]

Supplementary information

on

Optimizing laser-driven proton acceleration from overdense targets

A. Stockem Novo, M. C. Kaluza, R. A. Fonseca and L. O. Silva

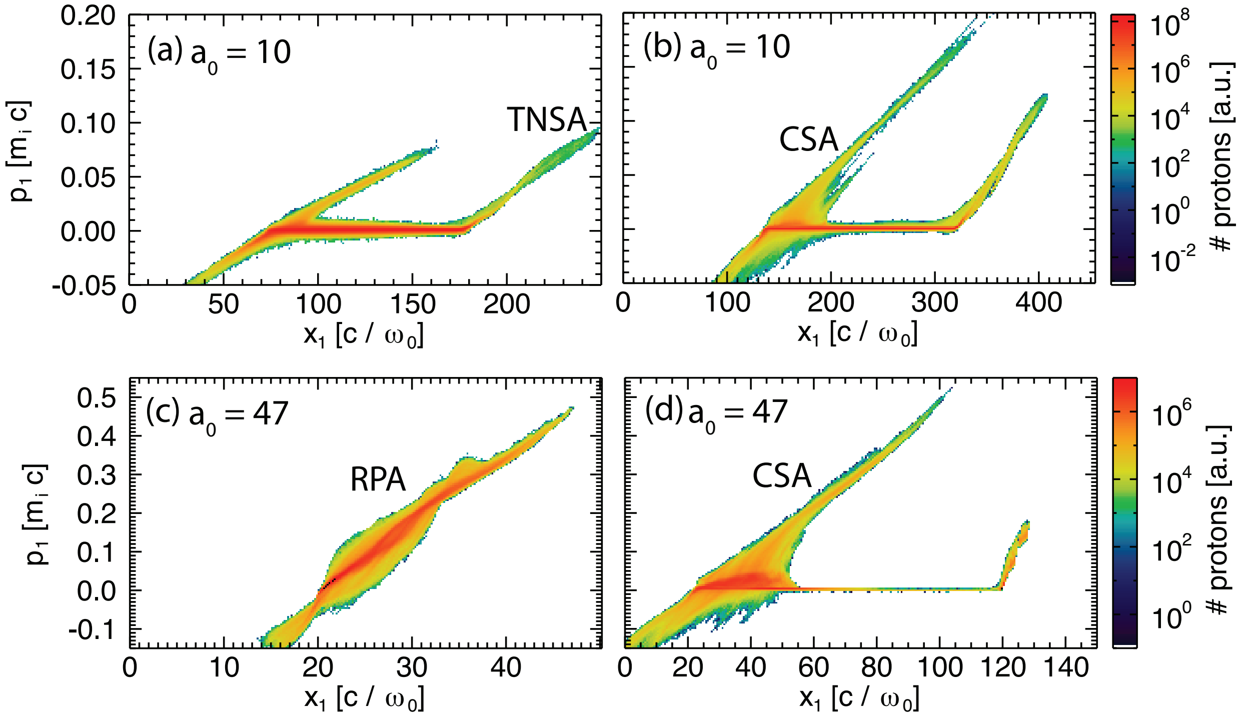

FIG. S1: Proton phase spaces for $a_0 = 10$ at time $t = 0.8$ ps for representative cases TNSA $x = 4$ (a) and CSA $x = 0.67$ (b) and for $a_0 = 47$ for RPA $x = 13$ at $t = 0.07$ ps (c) and CSA $x = 0.5$ at $t = 0.12$ ps.

In the pure TNSA-regime $n_0/\gamma n_{cr} > 2$, the associated electric field is not strong enough to accelerate the ions inside the target and acceleration occurs mainly at the target rear surface (see Fig. S1a).

In the case of near-critical densities $0.5 \leq n_0/\gamma n_{cr} \leq 2$ a strong peak of the electric field picks up the ions and accelerates them due to collisionless shock acceleration (Fig. S1b, d). The bunch of accelerated particles can be clearly identified in the ion phase space where collisionless shock acceleration at the target front dominates over TNSA at the target rear surface.

In the case of radiation pressure acceleration $n_0/\gamma n_{cr} \gg 1$ the target is destroyed and almost all the ions are accelerated (Fig. S1c).

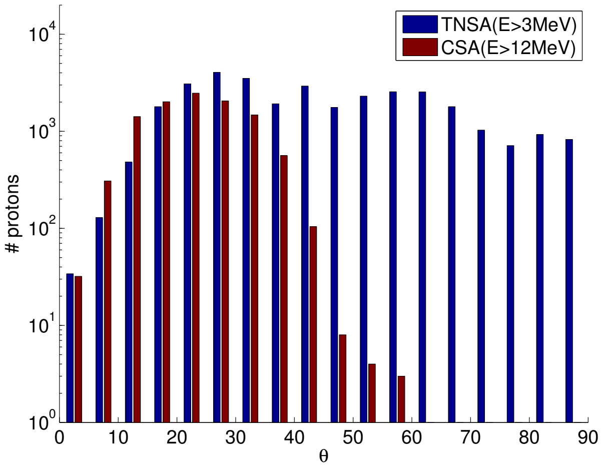

FIG. S2: Number of protons per $\Delta\theta = 5°$ in case of TNSA (blue) with ion energies above 3 MeV for $n_0/\gamma n_{cr} = 1$ and CSA (red) above 12 MeV for $n_0/\gamma n_{cr} = 0.67$ at $t = 0.8$ ps for a cubic target.

Fig. S2 shows the advantage of collisionless shock acceleration (CSA) towards target-normal-sheath acceleration (TNSA). The TNSA case shows an almost uniform distribution of the protons while the CSA case shows a narrow distribution at low angles.
